# Diversity patterns, ecological associations and future of research on Costa Rican myxomycetes

**DOI:** 10.1080/21501203.2018.1481153

**Published:** 2018-06-05

**Authors:** Carlos Lado, Carlos Rojas

**Affiliations:** a Department of Mycology, Real Jardín Botánico (RJB, CSIC), Madrid, Spain; b Forest Resources Unit, Engineering Research Institute, University of Costa Rica, San Pedro de Montes de Oca, Costa Rica; c Experimental Interdisciplinary Station of Agroecological Models (FEIMA), University of Costa Rica, Turrialba, Costa Rica

**Keywords:** Amoebozoa, biodiversity, Central America, conservation, database, management, Mesoamerica, microorganisms, Myxogastria, slime molds

## Abstract

The most active research period on myxomycetes in Costa Rica has taken place in the last three decades. During this time, most of the collections have been carried out and most of the scientific articles have been produced. However, the lack of standard protocols and systematic planning across the country generated a need to conduct an analysis of myxomycete records to define future lines of work. A compilation, cleaning, standardisation and analysis of information associated with a database of more than 7800 records that comprised 242 species of myxomycetes reported in Costa Rica during the last 110 years, was carried out. An interpretation of data with a conservation approach that integrated elements of data-mining and geographical information systems was conducted. Results showed that myxomycetes has been comparatively well studied in Costa Rica in relation to other regional or tropical countries. However, survey effort has been unequal within the territory, leaving some interesting areas or substrates understudied. The absence of long-term goals to study this country and Mesoamerica has limited the potential that the analysed data can have within the context of conservation. This could be the next logical step in the study of this group of microorganisms in that country.

## Introduction

Large-scale biodiversity studies with emphasis on biogeographical elements are required for several groups of microorganisms (see Fontaneto and Brodie 2011). However, robust datasets accumulated over time are not available for most of these groups in most parts of the world. In the Mesoamerican context, Costa Rica is a unique study case for myxomycete analysis given its research history and development of investigations (Rojas and Doss 2013). These efforts may have wider geographical implications since the regional baseline knowledge (see Cotterill et al. 2013) has been accumulated in that territory.

Costa Rica is a country within the Mesoamerican Biodiversity Hotspot (Brummitt and Lughadha 2003) with a land surface of 51,100 km^2^ and an elevation range between sea level and 3820 m. Its biodiversity has been comparatively more studied than other regional countries due to a combination of political and historical elements as well as infrastructural and academic characteristics (see Evans 1999). This country has been used historically as a benchmark to establish comparisons of tropical biodiversity (see Janzen 1988). However, such artefact of research, product of the availability of high-quality biological datasets, may simply reflect the early establishment and continuous operation of world-class biological stations in the forests of Costa Rica.

As a logical consequence of the latter, some regions of the country have been over studied in comparison with others, thus affecting the generation of objective comparative systems of biodiversity assessment for administrative or ecological units, which rely on more standard efforts in each of these individual elements. This is not only a problem in Costa Rica, and Gotelli and Coldwell (2001) mentioned that most of the relative comparisons of biodiversity among countries, regions, ecosystems or biomes are constrained by methodological issues that limit the actual application of such evaluations. In the case of myxomycetes, for instance, comparisons of diversity across Central American countries do not reflect natural patterns and are simply the product of sampling schemes. For instance, the study of myxomycetes in Nicaragua, Costa Rica and Panama started in the early part of the 1900s, contrasting dramatically with incipient myxomycete research in El Salvador or Belize, which started in the last 20 years (see Lado and Wrigley de Basanta 2008; Rojas et al. 2013).

Interestingly, even within a country like Costa Rica, well studied in terms of vertebrate and plant biodiversity, the effort placed to study groups like the myxomycetes has been far from being evenly distributed across the territory. For instance, Schnittler and Stephenson (2000) carried out a reference study for ecological purposes but mainly focused on tropical dry areas of the northwestern section of the country, thus leaving the rest of the territory understudied. This uneven distribution of work is not surprising, and it demonstrates that for some microscopic groups it is difficult to establish organised systems of study due to the comparatively low numbers of researchers than for macroscopic organisms. This constraint has obvious effects on conservation efforts, which rarely include microorganisms in the agenda.

Despite the latter, for myxomycetes, Costa Rica does not represent a weak model of integrated study either. Work has been carried out since the 1970s, when Alexopoulos and Sáenz (1975) and Farr (1976) started systematising research in this territory. Consequently, the compilation of information and integrated analysis of historical research as well as the determination of future lines of study for Costa Rica has lagged the strategies determined for other groups of organisms, but it is ahead of other tropical countries.

The present study aimed at the construction of a nomenclatural and ecological database with information on myxomycetes from Costa Rica as a strategy to promote the concept of standardisation within a conservation framework with a focus on this group of microorganisms. This is strategic due to the importance of biodiversity research within a context of achievable taxonomic goals using standard protocols for prioritising conservation units (see Margules et al. 2002). However, such task is only possible through deep analysis of information, which is performed using data-mining and pattern visualisation techniques. For countries with a recent history of myxomycete study like Costa Rica, this is very important to provide an answer to logical questions of applied biological research based on previous research patterns. Such consolidation of a standardised database also promotes educational and informational components, which aid to close the bioliteracy gap on microscopical organisms.

## Materials and methods

This project was carried out during 2016 and 2017 both in the Royal Botanical Gardens of Madrid, Spain and the Engineering Research Institute of the University of Costa Rica, in the framework of a cooperation research grant (COOPB20155) funded by the Spanish Research Council.

A consolidated database of myxomycete records for Costa Rica was compiled from several sources. These included the herbarium of the National Museum of Costa Rica (acronym CR), the herbarium of the University of Costa Rica (USJ) and the Farlow Herbarium in Harvard University (FH). Additionally, data from the personal collection of Martin Schnittler (deposited at Botanische Staatssammlung München, Germany, acronym M), the myxomycete collection at the University of Arkansas (abbreviated as UARK) and the Myxogastrid Biorepository of the Engineering Research Institute at the University of Costa Rica (abbreviated as INII) were used. These are the collections in the world with most of the myxomycete records from Costa Rica.

All actual vouchers deposited at CR, USJ and INII were checked individually in the respective herbarium. Information of collections from UARK and M were checked online and cross-validated with personal databases from the collectors (Steven L Stephenson at UARK, Martin Schnittler at M). Data from FH were checked online in the database of the herbarium. All collections of myxomycetes were cross-checked with a previous database constructed by Rojas et al. (2010) and the final database was compared, looking for inconsistencies, with a database of bibliographic records from the Neotropics constructed by the Myxotropic project (www.myxotropic.org).

The final database contained information such as herbarium and collector number, species name, date of collection, province, county and locality information, georeference, elevation, forest type, climate zone information, substrate, collector and identifier. After the consolidation of this unique database, all collecting dates were revised and corrected to a standard format to eliminate inconsistencies. For the geographical work, the independent administrative units known as “cantones” in Costa Rica have been referred to as counties in this study. Criteria for georeferencing included the use of decimal coordinates and the WGS84 datum. Georeferences were assigned to all collections either using the direct information from collectors, obtained with a personal GPS unit, or by establishing centroids associated with localities where collections were made. In the second case, to minimise possible errors, areas with average elevation within county of collection were used to select centroids and additional information in the Herbarium USJ was retrieved from original collecting logbooks. All other missing elevations were assigned to records in a similar manner (using geographical centroids for the most accurate location assigned). For substrates, a series of categories such as bark and wood, ground litter, aerial litter, flowers and inflorescences, fruits, lianas, living cryptogams, living plants, twigs and dung were created and used to standardise the database. These categories are based on the microhabitat separation defined by Rojas et al. (2014). Bark and wood is the equivalent of coarse woody debris and living cryptogams is the substrate associated with epiphyll myxomycetes. Records associated with living plants were separated from the latter. In the case of all variables standardised in the database, original information either from the physical voucher labels or from databases was maintained for reference purposes. Similarly, all collecting numbers assigned by collectors and herbarium numbers assigned when vouchers were deposited were maintained and cross-checked among partial databases. Nomenclature for myxomycetes was based on Lado (2005-2017), forest types were named according to Holdridge (1967) with modifications by Ortiz-Malavassi (2014) and conservation areas are named in accordance with local legislation.

After that process, a series of analyses were carried out on the database with the objective to summarise the information and examine patterns. For this part of the study, data-mining techniques were carried out by creating data flow analysis and visualisation nodes on KNIME v.3.4 (Berthold et al. 2008). Summarised data from the previous step were either used to create maps on QGIS v.2.18 (QGIS Development Team 2004-2014) or analysed statistically on JMP v.10 (SAS Institute 1989-2007). Diversity indices and calculation of ACE and iChao 1 (improved Chao 1) values for the maximum number of species were performed using Spade in R (Chao et al. 2015). The second one was selected based on Chiu et al. (2014). Rarefaction curves and diversity profiles were created with iNEXT for R (Hsieh et al. 2016) based on Chao et al. (2014) for both the consolidated database constructed herein and a previous one based on Rojas et al. (2010). For table creation, species selection was performed considering only those species with dissimilar diversity profiles. Statistical differences observed in contingency analyses using an alpha of 0.05 were shown in the tables as bolded values.

## Results

**Figure 1.**
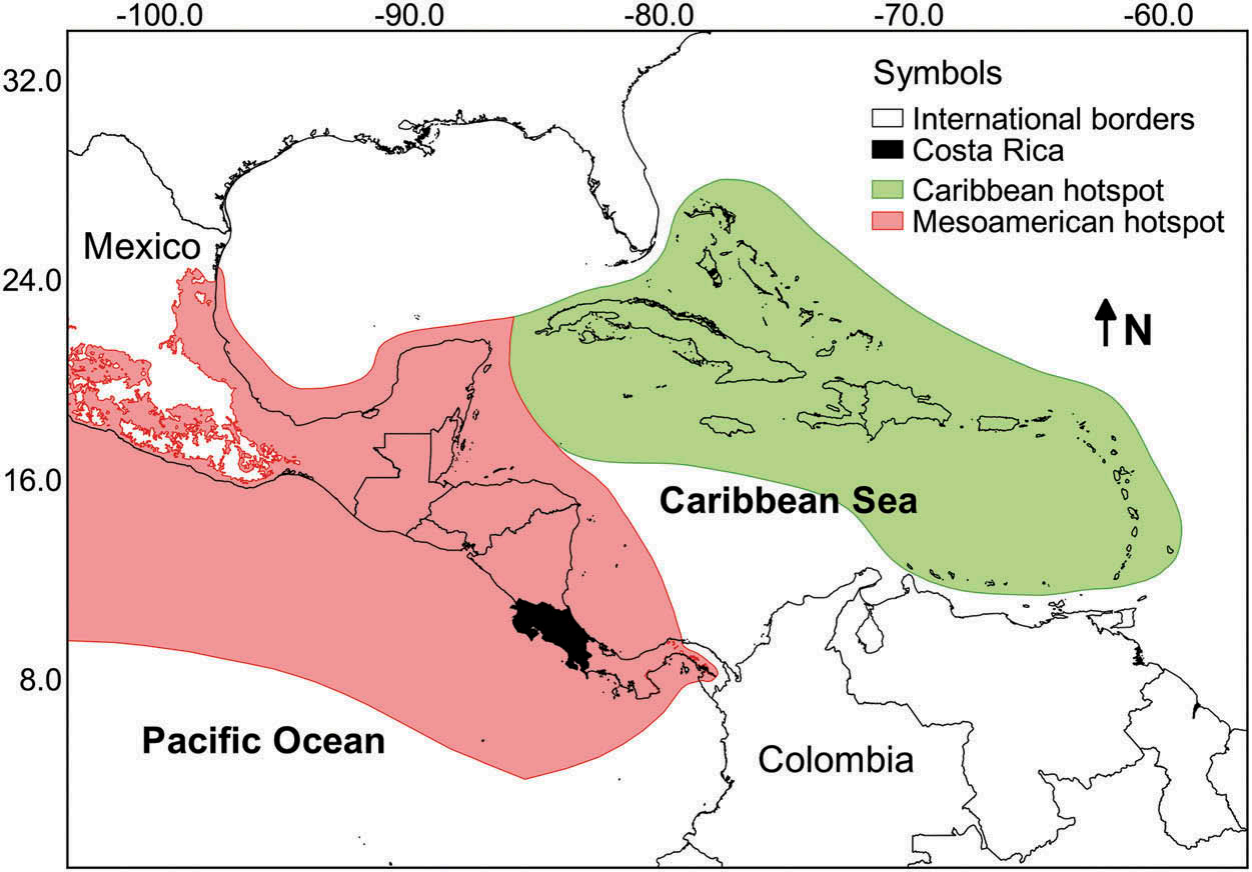
Map of the central section of the Americas showing the relative location of Costa Rica along with the Caribbean and Mesoamerican biodiversity hotspots.

**Figure 2.**
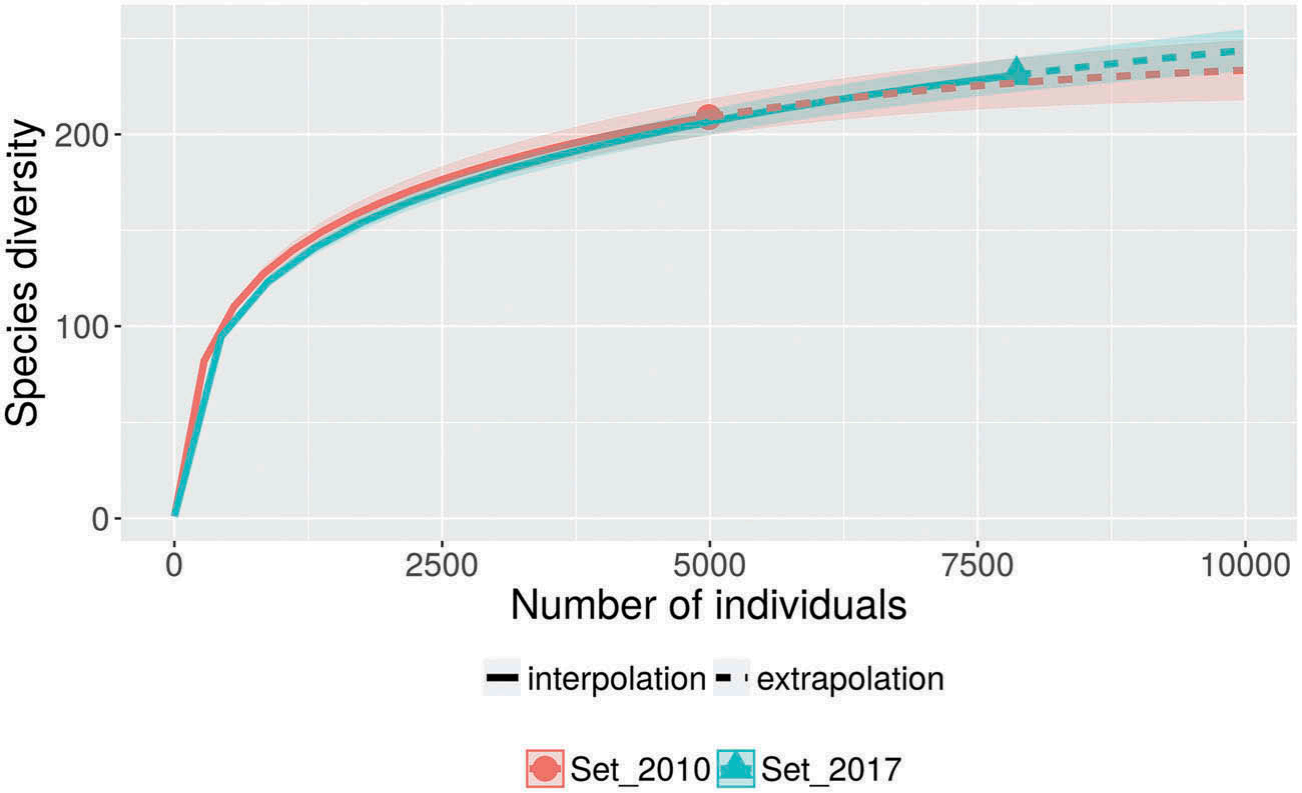
Rarefaction curves for the complete dataset considered herein (Set_2017) and a partial dataset considering records made until 2010 (Set_2010), showing the confidence intervals as shaded areas along the main line and both the inter- and extrapolation sections with an estimation of 10,000 records.

**Figure 3.**
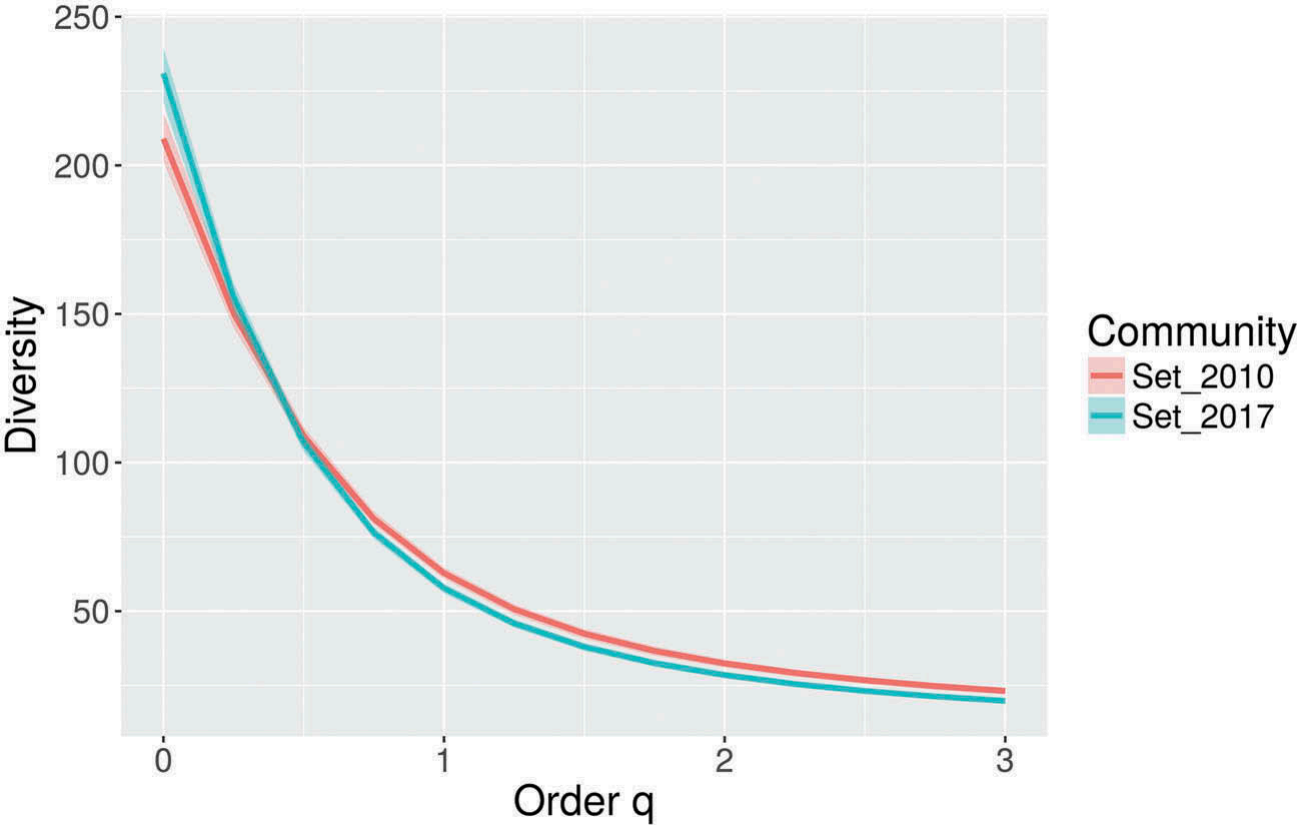
Hill number-based diversity profiles for both the complete dataset considered herein (Set_2017) and a partial dataset considering records made until 2010 (Set_2010).

A database with more than 7800 records of myxomycetes in Costa Rica was compiled. This dataset contained 242 species in 40 genera, providing further evidence of the richness in the Mesoamerican biodiversity hotspot (see Figure 1). These values represent around 25% of the myxomycete species known in the world, more than double the known species in Cuba, the best-known country in the Caribbean biodiversity hotspot, and more than 50% of the species recorded by Lado and Wrigley de Basanta (2008) for the Neotropics. The ACE and iChao 1-estimator values for the maximum number of species based on the current dataset were 274 and 295 species, respectively. The range of possible number of species to be expected in the country based on the 95% confidence intervals for the iChao 1 estimator was calculated between 274 and 325 species. The taxonomic diversity index (TDI) was calculated as 5.95 and the overall Shannon’s and 1-D Simpson’s diversity indices were 4.05 and 0.96, respectively. When compared with a previous record dataset from 2010 (Rojas et al. 2010) when the TDI value was 5.77, results showed rarefaction curves to be similar (Figure 2). This demonstrates that the previous effort of 4990 records had already explained in detail the pattern of increasing number of species to be expected (extrapolation on Set_2010). In fact, with a 50% increment in sampling effort (up to 7500 records), the current dataset (Set_2017) generated a very similar curve within the boundaries provided by the 95% confidence intervals of the first one. In an analogous manner, the diversity profiles of both sets (Figure 3) were very similar and showed the current dataset to be associated with higher species richness (associated with *q* = 0) but lower diversity index values (*q* > 1).

**Figure 4.**
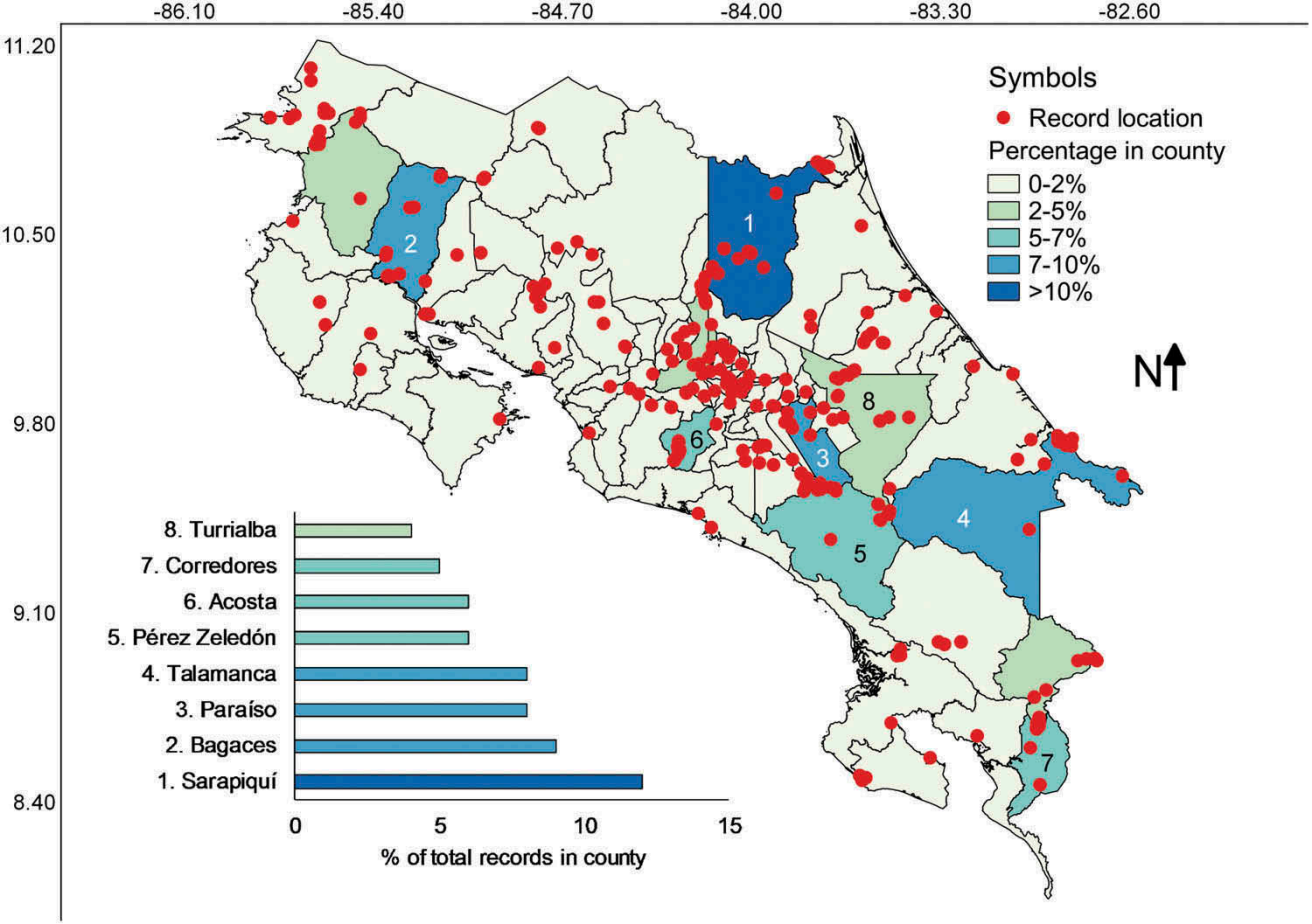
Map of Costa Rica showing the location of myxomycete records within the boundaries of administrative counties coloured according to record frequency. The eight counties with most records are shown at the bottom of the map.

Records were found in all seven provinces of Costa Rica but only in 64 of the counties, which represented 78% of the 82 administrative units in the country (see Figure 4). The most studied counties were Sarapiquí, Bagaces, Paraíso and Talamanca, coinciding with strong research carried out by Steven Stephenson, Martin Schnittler and the second author of this study in the localities of La Selva Biological Station, Palo Verde National Park, Cerro de la Muerte and Cahuita. The only counties with no records formally assigned to them were: Abangares, Alajuelita, Cañas, Esparza, Flores, Guácimo, Guatuso, Matina, Moravia, Nandayure, Naranjo, Orotina, Palmares, Río Cuarto, San Pablo, Tilarán, Valverde Vega and Zarcero. Interestingly, some of these are among the counties with the lowest forest cover in Costa Rica. Table 1 shows a summary of counties with numbers of records and species associated with them according to the database built; and the Pearson’s correlation value between county area size and number of records was 0.46 whereas with number of species it was 0.47, showing moderate to weak relationships.

**Table 1.** Summary of county size information and recorded myxomycetes according to the dataset compiled in the present investigation.

County	Area (km^2^)	Total area (% total)	Estimated forest cover (%)	Myxomycete records	Myxomycete species
Alajuela	388.4	0.76	30	4	4
San Mateo	125.9	0.25	30	5	5
San Carlos	3347.9	6.55	25	11	7
San Ramón	1018.6	1.99	45	13	8
Los Chiles	1358.8	2.66	10	11	9
Atenas	127.1	0.25	30	28	11
Poás	73.8	0.14	20	30	11
Upala	1580.6	3.09	30	55	19
Grecia	395.7	0.77	30	227	19
El Guarco	167.6	0.33	45	2	2
La Unión	44.8	0.09	30	2	2
Alvarado	81.0	0.16	30	5	3
Cartago	287.7	0.56	45	34	16
Oreamuno	202.3	0.40	15	33	16
Jiménez	286.4	0.56	60	108	22
Turrialba	1642.6	3.21	70	462	47
Paraíso	411.9	0.81	65	532	63
Hojancha	261.4	0.51	45	1	1
Carrillo	577.5	1.13	45	3	2
Santa Cruz	1312.2	2.57	65	7	6
Liberia	1436.4	2.81	45	91	14
Nicoya	1333.6	2.61	45	119	17
Bagaces	1273.4	2.49	45	634	78
La Cruz	1383.9	2.71	65	414	89
Belén	12.1	0.02	15	2	2
Santa Bárbara	53.2	0.10	15	3	3
San Isidro	26.9	0.05	25	5	4
Heredia	282.6	0.55	45	22	9
Barva	53.8	0.11	25	35	12
Santo Domingo	24.8	0.05	15	57	15
San Rafael	48.3	0.09	25	28	17
Sarapiquí	2140.5	4.19	50	1071	97
Limón	1765.7	3.46	70	5	4
Siquirres	860.1	1.68	70	34	15
Pococí	2403.4	4.70	30	91	22
Talamanca	2809.9	5.50	80	729	66
Garabito	316.3	0.62	70	1	1
Montes de Oro	244.7	0.48	50	1	1
Golfito	1753.9	3.43	50	5	4
Aguirre	543.7	1.06	50	5	4
Buenos Aires	2384.2	4.67	50	123	22
Osa	1930.2	3.78	50	95	23
Parrita	478.7	0.94	30	278	32
Coto Brus	933.9	1.83	50	155	51
Corredores	620.6	1.21	30	533	52
Puntarenas	1842.3	3.61	45	566	89
Coronado	222.2	0.43	50	1	1
Escazú	34.4	0.07	30	1	1
Curridabat	15.9	0.03	15	1	1
Goicoechea	31.5	0.06	30	2	2
Tibás	8.1	0.02	15	2	2
Aserrí	167.1	0.33	50	4	3
Tarrazú	297.5	0.58	40	4	4
Desamparados	118.2	0.23	30	4	4
León Cortés	120.8	0.24	30	7	6
Turrubares	415.2	0.81	70	17	7
Mora	162.0	0.32	50	13	10
San José	44.6	0.09	15	37	12
Santa Ana	61.4	0.12	50	53	17
Puriscal	553.6	1.08	50	119	20
Acosta	342.2	0.67	50	179	26
Dota	400.2	0.78	40	108	40
Montes de Oca	15.1	0.03	30	132	47
Pérez Zeledón	1905.5	3.73	50	497	84

Cartago was the only province that showed myxomycete records associated with all its counties. Puntarenas and San José had over 90% of their counties studied, Heredia about 80%, Limón and Guanacaste over 64% and the least studied province was Alajuela with only 53% of its counties showing records of myxomycetes. Interestingly, the correlation between the county-based completeness and the relative frequency of records per province was 0.69, showing that within provinces some counties were partially over studied in comparison with others.

**Figure 5.**
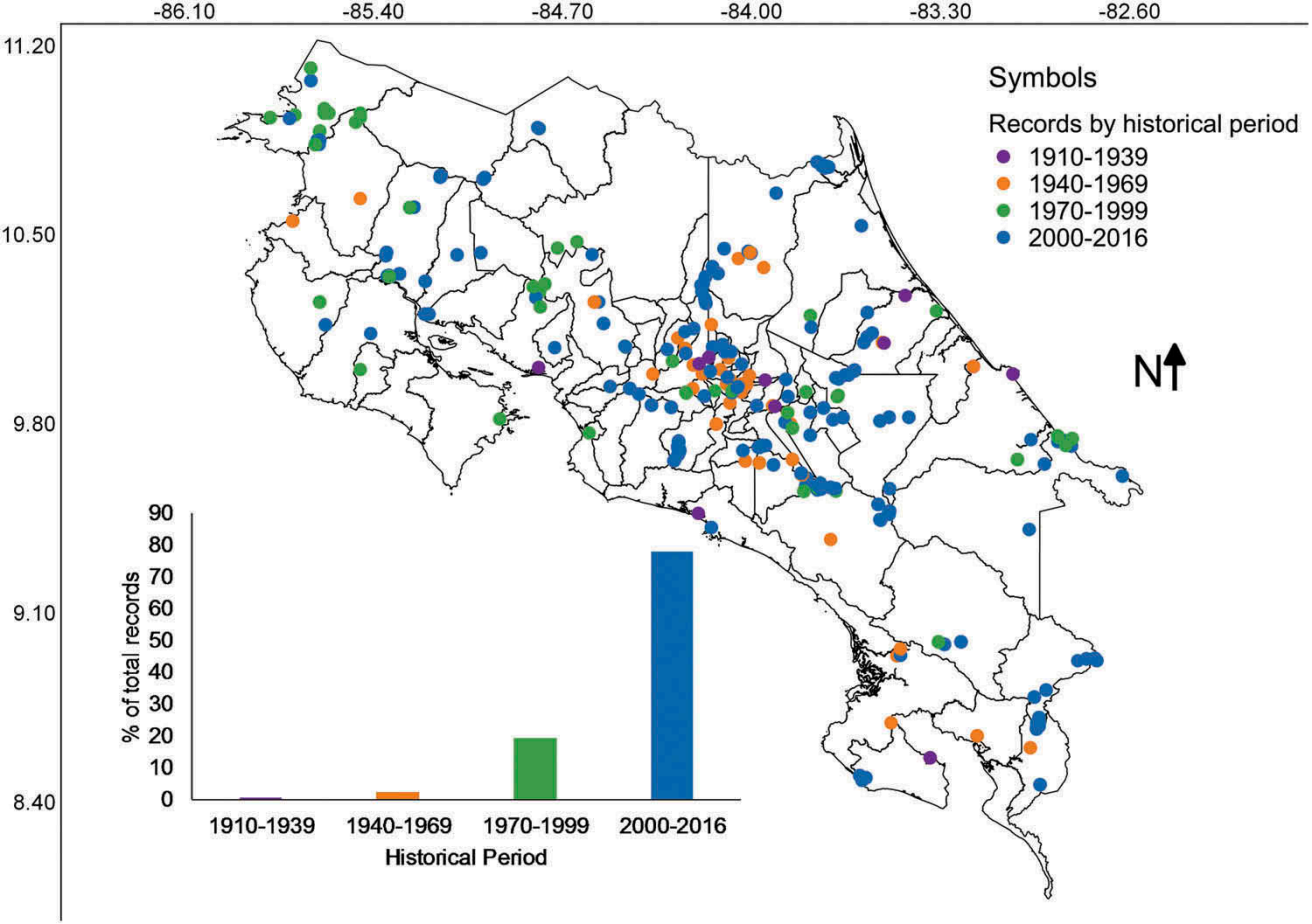
Map of Costa Rica showing the location of myxomycete records according to period when collections were made depicted as coloured categories. The relative frequency of records over time is shown at the bottom of the map.

The distribution of records by geographical location and historical period within the last 106 years is shown in Figure 5. It was clear that most of collections and the broader distribution of collecting locations are associated with the last three decades. Also, the central part of Costa Rica seems still to be an important region for myxomycete research. It is interesting to note that the period with myxomycete research backed up by deposited collections started in the 1960s, when the North American researcher Constantine J. Alexopoulos teamed up with local professor José Alberto Sáenz. Both researchers collected in several locations and produced the first consolidated list of Costa Rican myxomycetes (Alexopoulos and Sáenz 1975).

**Figure 6.**
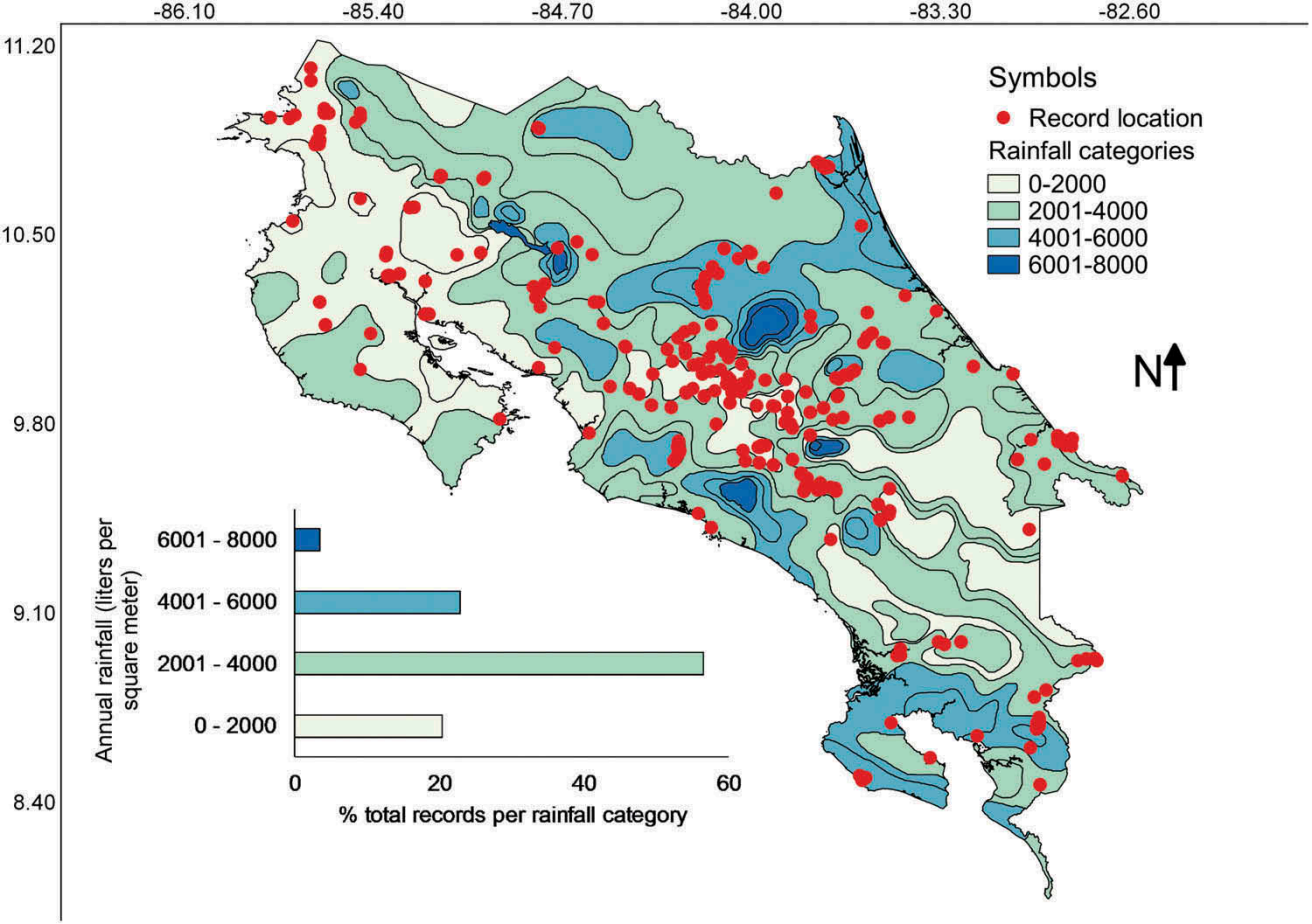
Map of Costa Rica showing the location of myxomycete records within the precipitation zones. The distribution of records associated with precipitation categories is shown at the bottom of the map.

**Figure 7.**
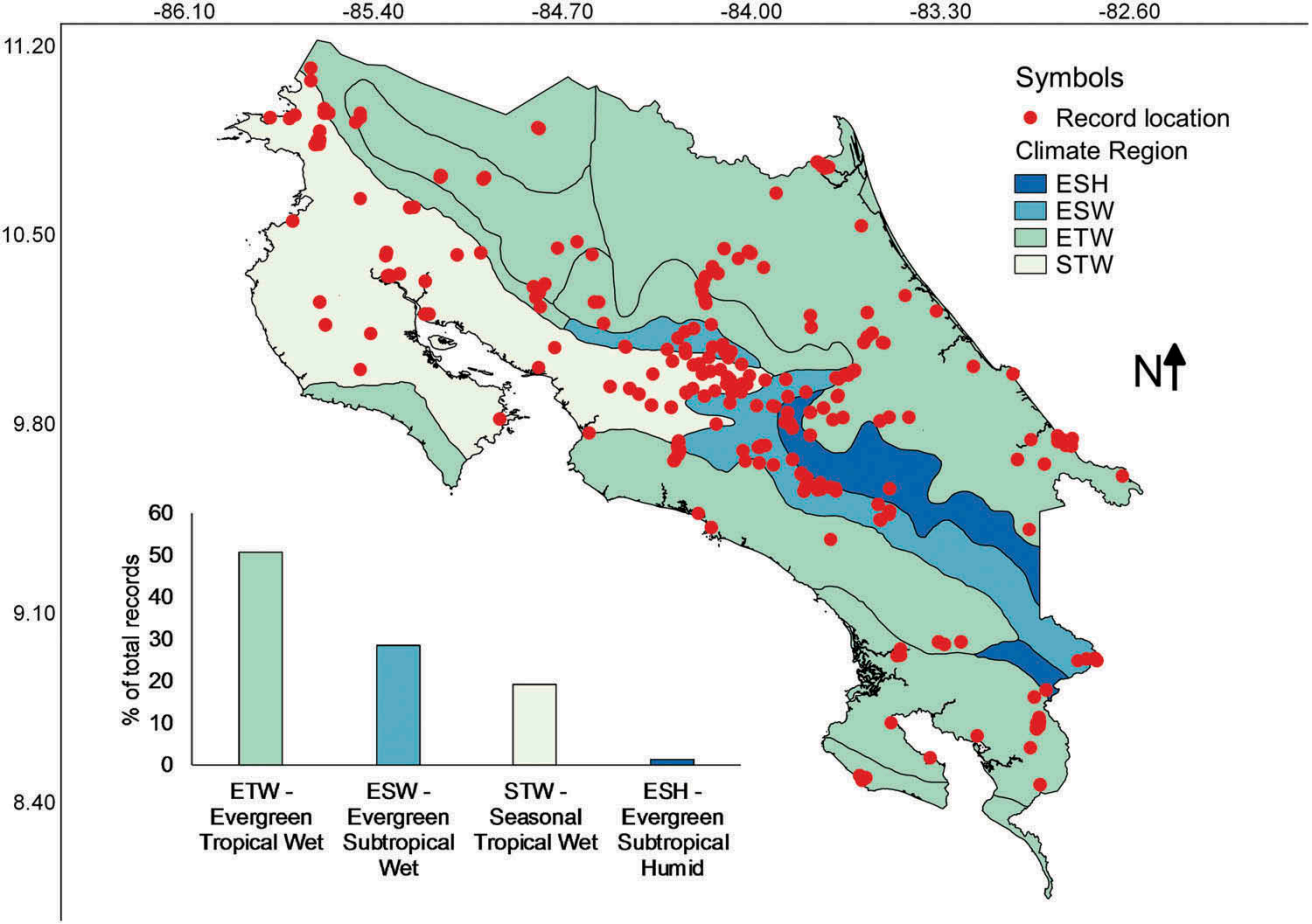
Map of Costa Rica showing the location of myxomycete records within climate regions. The distribution of records associated with these regions is shown at the bottom of the map.

Most collections have been made in areas with an average precipitation between 2000 and 4000 L/m^2^ (Figure 6) corresponding with the Evergreen Tropical Wet forest type (Figure 7). Few collections (relatively speaking) have been made in areas with precipitation higher than 6000, lower than 1500 L/m^2^ (equivalent to mm) or within the evergreen subtropical humid forest type. A series of Pearson’s correlations calculated between the number of collections from each category of precipitation or forest climate region and the relative area of the country represented in each category resulted in a high *r* value of 0.91 and 0.85 for precipitation and forest climate regions, respectively. The latter suggested that relative frequency of myxomycete records was dependent on the area size associated with both precipitation categories and forest climate regions, showing that all categories have been surveyed adequately. Interestingly, the intuitive Simpson’s index of diversity (1-D) did not follow the same pattern for precipitation (*r* = 0.52), suggesting that both variables are independent of each other and that some species may be associated with dryer or wetter regions.

Table 2 summarises the number of records for some species of myxomycetes found to be associated with precipitation categories and demonstrates that *Arcyria incarnata, Diachea bulbillosa, Lycogala exiguum* and *Tubifera microsperma* have primarily been recorded in dryer areas. Similarly, other species such as *Craterium concinnum, Cribraria mirabilis, Diachea leucopodia, Leocarpus fragilis* and *Willkommlangea reticulata* were found to be associated with wetter regions. Table 3 shows that most record associations with a geographical climate region were observed for the pacific coast, particularly the South Pacific. Interestingly, *Stemonaria gracilis* has only been observed in the south Caribbean and *Cribraria costata* as well as *Trichia persimilis* showed a strong association with the central valley.

**Table 2.** Numbers of records for selected species of myxomycetes showing associations (in bold) with precipitation categories according to the database constructed herein.

	Annual average Precipitation (L/m^2^)			
Myxomycete species	1500–2000	2000–3000	3000–4000	4000–5000
*Arcyria incarnata*	**16**	2	7	
*Arcyria insignis*	8	**35**	7	
*Ceratiomyxa morchella*	**6**		1	1
*Ceratiomyxa sphaerosperma*			**3**	**3**
*Craterium concinnum*	2		**14**	
*Cribraria costata*		**7**		
*Cribraria mirabilis*			**18**	
*Cribraria purpurea*	1		**19**	
*Diachea bulbillosa*	**9**		1	
*Diachea leucopodia*	3	3	**27**	11
*Diderma chondrioderma*			**5**	
*Lamproderma columbinum*			**12**	
*Lamproderma muscorum*	**7**			
*Leocarpus fragilis*			**15**	
*Lycogala exiguum*	**14**	1		
*Macbrideola scintillans*	**11**			1
*Physarum javanicum*	**6**	1	1	1
*Physarum serpula*	2	**30**		2
*Physarum superbum*	5	**26**	4	3
*Stemonaria gracilis*		**13**		
*Trichia botrytis*		2	**11**	
*Trichia decipiens*	1	5	**11**	
*Trichia favoginea*		9	**24**	
*Trichia persimilis*		**12**	1	
*Trichia verrucosa*		4	**10**	
*Tubifera microsperma*	**26**	1	3	
*Willkommlangea reticulata*			**9**

**Table 3.** Numbers of records for selected species of myxomycetes showing associations (in bold) with geographical climate regions^a^ according to the database constructed herein.

	Geographical climate region							
Myxomycete species	NC	SC	CI	CP	NP	SP	CV	HN
*Arcyria magna*							**3**	
*Cribraria costata*							**7**	
*Cribraria mirabilis*						**18**		
*Cribraria piriformis*						**3**		
*Cribraria purpurea*					**1**	**19**		
*Diachea bulbillosa*					**9**	**1**		
*Diderma chondrioderma*						**5**		
*Didymium listeri*					**7**			
*Lamproderma columbinum*						**12**		
*Lamproderma echinulatum*						**4**		
*Lamproderma magniretisporum*						**3**		
*Lamproderma muscorum*					**7**			
*Lamproderma sauteri*						**2**		
*Lycogala exiguum*					**14**		**1**	
*Macbrideola martinii*					**17**			
*Stemonaria gracilis*		**13**						
*Stemonitopsis aequalis*					**12**		**3**	
*Trichia persimilis*				**1**			**12**	
*Trichia verrucosa*						**10**	**4**	

^a^ Abbreviations: NC: North Caribbean; SC: South Caribbean; CI: Cocos Island; CP: Central Pacific; NP: North Pacific; SP: South Pacific; CV: Central Valley; HN: Huetar North

**Figure 8.**
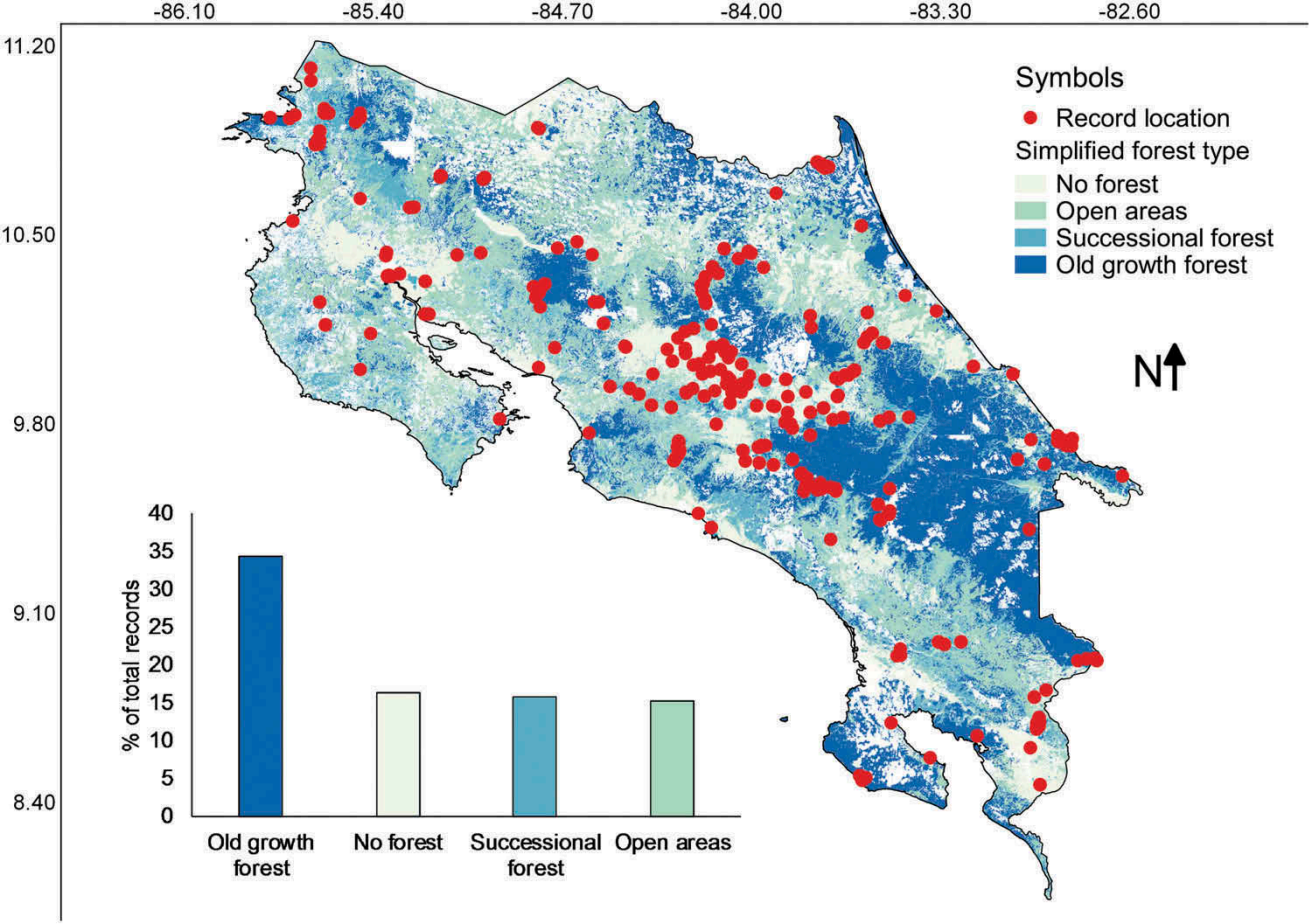
Map of Costa Rica showing the location of myxomycete records within a simplified scheme of forest type categories. The distribution of records associated with these categories is shown at the bottom of the map.

Most of the records were found in old growth forests across the country (Figure 8). Non-forested areas, successional forests and open areas accounted all together for about 50% of the records. Pearson’s correlations calculated between the number of collections from each category of forest type and the relative area of the country represented in each category resulted in an *r* value of 0.91. In a similar manner to previous cases, such a high value suggests that record frequency is associated with the area size of forest type categories.

**Figure 9.**
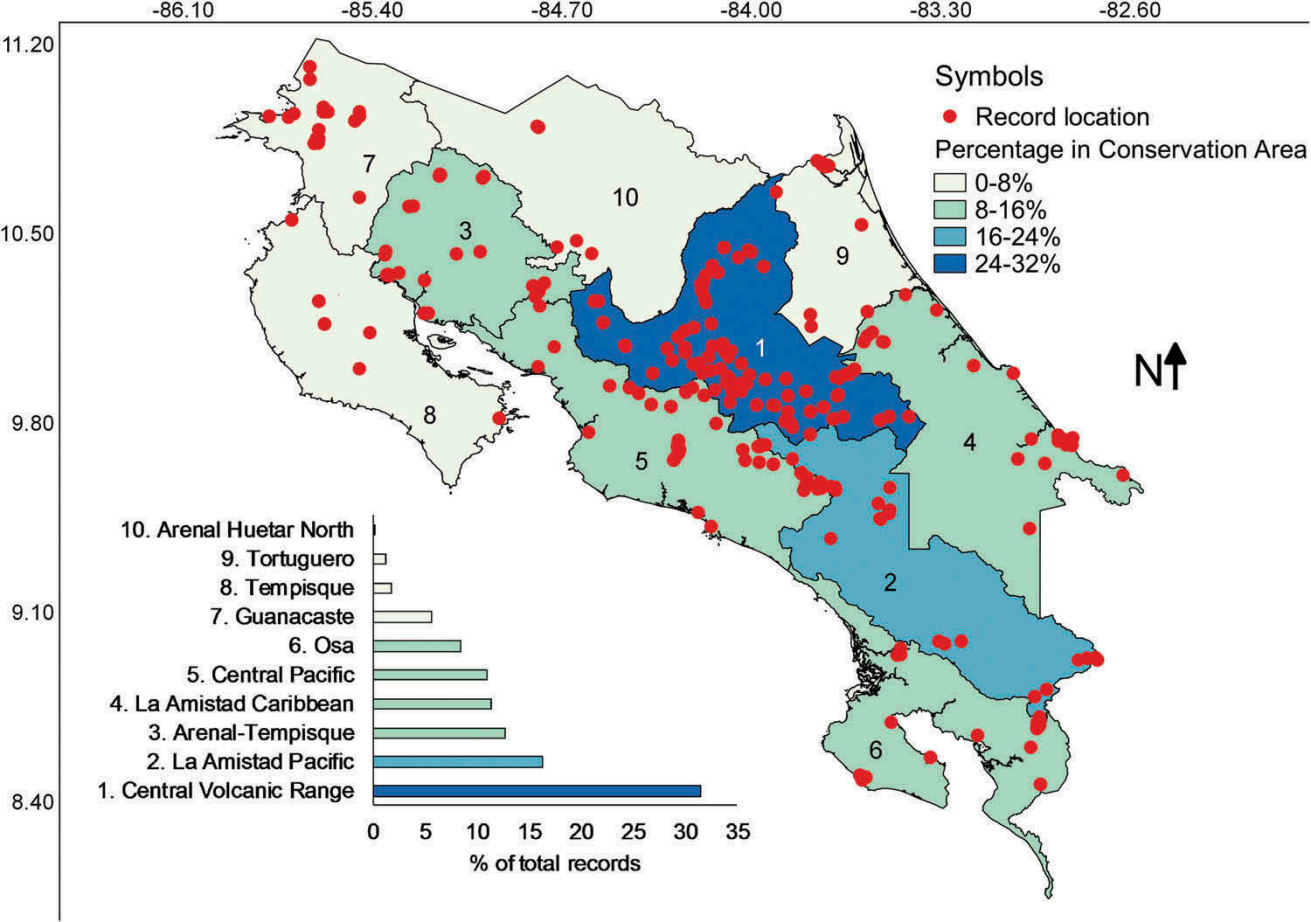
Map of Costa Rica showing the location of myxomycete records within the boundaries of conservation areas coloured according to record frequency. The distribution of records associated with these areas is shown at the bottom of the map.

When the conservation areas of Costa Rica were used to analyse data, results showed that all conservation areas have been surveyed and that the Central Volcanic Range is where over a third of the records have been collected (Figure 9). Following the latter, La Amistad-Pacific, Arenal-Tempisque and La Amistad-Caribbean accounted for 40% of all records. The least studied conservation areas were Arenal-Huetar North and Tortuguero. In this case, Pearson’s correlation between the number of records and the area encompassed by each conservation area gave an *r* value of 0.43 but did not result in significant differences indicating independence between variables (*P* = .20). This result suggests that sampling intensity has been unequal across conservation areas as can be inferred from the fact that the Arenal-Huertar North Conservation Area has the largest percentage of country area with 13% but was only associated with 0.5% of the myxomycete records.

The intuitive Simpson’s index of diversity (1-D) showed a weak correlation with conservation area size (*r* = 0.31), suggesting independence of variables and indicating that some species could have regional specificity. Table 4 shows that the two conservation areas with most species unique to a specific region were Amistad Pacific and Central Volcanic Range. It is interesting that a series of species of *Cribraria* and *Lamproderma* have only been recorded from the first one, whereas the second area showed unique species such as *Dictydiaethalium plumbeum, Physarum pezizoideum* and *Trichia persimilis*. The Central Pacific Conservation Area also showed some unique species such as *Didymium listeri* and *Lamproderma muscorum*.

**Table 4.** Numbers of records for selected species of myxomycetes showing associations (in bold) with conservation areas^a^ according to the database constructed herein.

	Conservation area										
Myxomycete species	A	B	C	D	E	F	G	H	I	J	K
*Alwisia morula*									**3**		
*Ceratiomyxa sphaerosperma*	1			**5**							
*Craterium concinnum*		**14**							2		
*Craterium paraguayense*									3		
*Cribraria mirabilis*		**18**									
*Cribraria piriformis*		**3**									
*Cribraria purpurea*		**19**							1		
*Dictydiaethalium plumbeum*				**3**							
*Diderma spumarioides*			**3**								
*Didymium listeri*									**7**		
*Lamproderma columbinum*		**12**									
*Lamproderma echinulatum*		**4**									
*Lamproderma magniretisporum*		**3**									
*Lamproderma muscorum*									**7**		
*Leocarpus fragilis*		**13**							2		
*Licea castanea*		**4**									
*Lycogala conicum*				**2**	**1**						
*Physarum leucophaeum*			2	**5**							
*Physarum pezizoideum*				**3**							
*Stemonaria gracilis*	**13**										
*Trichia persimilis*				**12**					1		
*Trichia verrucosa*		1		**4**							

^a^Abbreviations: A: Amistad Caribbean; B: Amistad Pacific; C: Arenal Tempisque; D: Central Volcanic Range; E: Guanacaste; F: Huetar North; G: Cocos; H: Osa; I: Central Pacific; J: Tempisque; K: Tortuguero

Finally, the distribution of records by substrates demonstrated that most myxomycetes in Costa Rica have been recorded in ground litter, bark and wood, flowers and inflorescences and aerial litter. These substrates accounted for 58%, 24%, 9% and 5% of all records with substrate information (about 96% of database). Lianas, fruits and dung were the least common substrates with less than 0.2% of records in all cases. With these results, most myxomycetes were associated with ground litter, bark and wood. However, particularly interesting was the case of *Physarum didermoides, Didymium bahiense, Physarum compressum* and *Perichaena dictyonema*, which were recorded on flowers and inflorescences 70%, 58%, 42% and 40% of the time, respectively. *Arcyria insignis* was recorded on living plants in a relative frequency of 40% and *Perichaena vermicularis* on aerial litter, about 30% of occasions.

## Discussion

The present study represents an update to the Costa Rican myxomycete review carried out by Rojas et al. (2010). All basic calculations associated with biodiversity increased in the present study in relation to the previous one. Even though such result is not surprising from an effort-based perspective, since about 2500 new records have been included in the general database during the last 7 years, it represents an interesting parameter to understand the effect of a sustained effort on general diversity patterns. For example, the calculated range for the maximum number of species increased in a logical manner due to an increased number of singletons in the database, represented by species recorded in the last years (e.g. Rojas et al. 2015). This result likely affected the calculation of diversity indices decreasing their value in relation to the reference dataset from 2010. However, as observed in Figures 2 and 3, the general diversity patterns between such reference and the present study did not differ dramatically, suggesting that most of the commonly observed morphological taxa had previously been recorded. In the context of Neotropical myxomycete research, these results represent a unique opportunity to understand the effect of sustained studies on biodiversity data. A further evaluation using molecular techniques would be extremely valuable to assess strengths and weaknesses of different techniques for different contexts, but the value of the data presented herein is the summarisation and standardisation of traditional myxomycete work for an entire country.

In general, the current dataset had an effect increasing all diversity-based calculations about 8% (from 3% for TDI to 12% for species richness) with an extra effort that represented 50% of the initial effort in 2010. This is important to mention since the current portrayal of myxomycete study in Costa Rica during the last years may represent an interesting study case under the scope of global ecological perspectives such as climate change, habitat disturbance and microbial distribution (e.g. Viscarra Rossel et al. 2014). In this context, for instance, previous regional datasets, such as those generated by Schnittler and Stephenson (2000) for Guanacaste, Monteverde and Cahuita or by Walker et al. (2015b) for La Selva Biological Station, are essential to determine diversity patterns over time. These areas are good candidates for future similar studies trying to determine changes in temporal arrangements. However, the execution of the present analyses also showed that some areas (see below) and forest types (i.e. mangroves, coastal forests) are undersampled. The latter is not only a problem in Costa Rica and may be a general issue in the northern section of the Neotropics. For this type of analyses, the use diversity profiles could be interesting to determine changes in different ecological estimators of diversity at different times. This type of figure has the potential to characterise the taxonomic, phylogenetic or functional diversity of an assemblage (see Chao et al. 2014).

When the record distribution is analysed in terms of counties, it is interesting to observe two patterns. First, most of the poorly studied or overseen counties have low percentages of forest cover in them (see Table 1). Second, as a consequence of the latter, the number of myxomycete records and species are independent of county size and province (also observed in Figure 4). These results show that most myxomycete research in Costa Rica has taken place in forested locations and has not been systematically arranged over spatial or temporal scales (also observed in Figures 5 and 8). Because of the latter, the distribution of substrates observed in the database may have also been skewed by limited sampling schemes. This is a logical result in the first phase of biological research, primarily dedicated to the documentation of species incidence and distribution. However, considering that most commonly recorded myxomycete morphospecies have been likely documented in this country, some additional efforts could simply focus research in a handful of locations, substrates and forest types, with a systematic approach, over moderately longer periods of time. This second step of biological research is also important from a perspective of natural resources management and environmental policy development (Bergstrom and Randall 2016). Independent of the latter, it is remarkable to observe that most myxomycete research has taken place in few counties (Figure 4) and only during the last decades (Figure 5).

When the analyses of records by precipitation zones and forest types were carried out, it was interesting but not surprising to observe that most collections have been made in areas with intermediate rainfall (Figure 6) in a common forest type such as the evergreen tropical wet forest in the country (Figure 7). This result can also be observed in Table 2. Since the study of myxomycetes in Costa Rica has been locality based and highly associated with national parks or forested areas, it is noteworthy to mention that even though most records are associated with areas sharing similar conditions, all forest types have been surveyed with an effort proportional to their relative area. In this manner, species associations with some climatic conditions suggest the patterns seem not to be an indirect product of unequal efforts in different climatic regions. As such, they seem to be real ecological associations, at least at the fruiting body and morphospecies level.

Another support to this idea comes from the results of the substrate analysis, which provided further evidence to the idea of an ecological guild of floricolous myxomycetes for tropical areas, as suggested by Schnittler and Stephenson (2002). One interesting aspect of this ecological association is that it seems not to be influenced as much by landscape-level features than by site-level forest characteristics (Rojas et al. 2017). In this sense, even though for the general analysis, the sampling of this type of substrate was proportionally lower than other ones such as wood and bark, results showed a high potential for hypothesis testing studies and conservation approaches. The latter is of course magnified within the context of the plant-rich Mesoamerican biodiversity hotspot.

In any way, according to Tables 3 and 4, an interesting region for further ecological analyses of potentially different assemblages of myxomycetes is the southern pacific, which has not received much attention in the past. The higher elevations in this part of the country (between 2000 and 3800 m) seem to host interesting myxomycetes with potential for biogeographical studies. At the regional level, some integrated studies in mangroves and costal ecosystems may provide interesting results for management purposes.

Independent of the fact that most collections have been made in forested areas, it is interesting to note that about 50% of all records were associated with non-forested areas, successional forests and open areas (Figure 8), supporting the idea of richness in non-forested conditions. Even though it is risky to establish such association due to normal changes in land use over time, it is likely that the actual areas where myxomycetes were collected actually corresponded with similar disturbed areas. This is due the fact that Costa Rica peaked in deforestation in the middle part of the 1980s (Sánchez-Azofeifa et al. 2001) and most collections considered in the present study were made after the middle part of the 1990s when disturbed areas were recovering.

The latter makes sense for most conservation areas but does not apply to Arenal-Huetar North, Tempisque and Tortuguero. Figure 9 shows these three to be the least studied areas in the country for myxomycetes. The first two have traditionally been agricultural regions where myxomycete researchers have done little work perhaps as a consequence of the difficulty finding accessible forest patches in a landscape dominated by private productive lands. However, the lack of myxomycete research in Tortuguero is intriguing since this is one of the most forested and pristine areas in the country. It is possible that logistic constraints and safety issues may account for these differences. At the regional level, there are also several locations (i.e. Gracias a Dios Department of Honduras, Amazonas Department in Colombia, Guayana Region in Venezuela), where few or none myxomycete studies have been carried out due to similar difficulties.

In any way, it seems imperative in this moment of myxomycete research in Costa Rica to equilibrate the effort by establishing surveys in the least studied regions in a similar manner to what has been done recently. In the partial dataset analysed in 2010 (Rojas et al. 2010), the Arenal Tempisque conservation area was understudied. This result triggered a moderately sustained effort in the last years, which equilibrated research in this area and made it the third most studied in the present study (Figure 9). A similar effort in the Tortuguero conservation area has already been initiated and it is expected to show similar results in the near future. Also, as mentioned before, research effort dedicated to understanding myxomycete incidence on poorly studied substrates (i.e. dung and lianas) or interesting ones (i.e. flowers and inflorescences) in Costa Rica is necessary, especially after evidence has shown the distinctive assemblages associated with some of them (i.e. Wrigley de Basanta et al. 2008).

In summary, the results from the present study show that myxomycetes in Costa Rica have been comparatively well studied in the last decades. At the regional and Neotropical level, these studies have generated important information to understand tropical myxomycete dynamics. The sustained effort to continue investigating their biological patterns at different scales of analysis, promoted in recent years, is already showing interesting results that can be used to analyse the ecological pressures affecting the reproductive stage of the group, at least in terms of morphological species. However, real systematic and sustained equal efforts in different ecological conditions would be the only manner to address several ecological questions. In this context, molecular information would be very useful for data interpretation. Ecological associations are an important element to contextualise and support biogeographical (i.e. Dagamac et al. 2017) or taxonomic (i.e. Walker et al. 2015a) studies and to promote applied lines of research on myxomycetes (i.e. Hoppe and Schnittler 2015). This type of information could be used in management plans and environmental impact assessments as a proxy to evaluate microorganisms. Also, under the current climate change and global land use change scenarios, this type of summarised studies represents important scientific inputs for the community of researchers studying patterns of microbial diversity change over time.
